# Building a precision oncology workforce by multidisciplinary and case-based learning

**DOI:** 10.1186/s12909-021-02500-6

**Published:** 2021-01-26

**Authors:** Srikar Chamala, Heather T. D. Maness, Lisa Brown, C. Brooke Adams, Jatinder K. Lamba, Christopher R. Cogle

**Affiliations:** 1 Department of Pathology, Immunology and Laboratory Medicine, College of Medicine, University of Florida, Gainesville, FL USA; 2 Center for Instructional Technology and Training, Information Technology, University of Florida, Gainesville, FL USA; 3 UF Health Cancer Center, University of Florida, Gainesville, FL USA; 4 Department of Pharmacy, UF Health Shands Hospital, Gainesville, FL USA; 5 Department of Pharmacotherapy & Translational Research, College of Pharmacy, University of Florida, Gainesville, FL USA; 6 Division of Hematology and Oncology, Department of Medicine, College of Medicine, University of Florida, 1600 SW Archer Road, Box 100278, Gainesville, FL 32610-0278 USA

**Keywords:** Case-based learning, Precision oncology, Graduate medical education

## Abstract

**Background:**

Participants in two recent National Academy of Medicine workshops identified a need for more multi-disciplinary professionals on teams to assist oncology clinicians in precision oncology.

**Methods:**

We developed a graduate school course to prepare biomedical students and pharmacy students to work within a multidisciplinary team of oncology clinicians, pathologists, radiologists, clinical pharmacists, and genetic counselors. Students learned precision oncology skills via case-based learning, hands-on data analyses, and presentations to peers. After the course, a focus group session was conducted to gain an in-depth student perspective on their interprofessional training experience, achievement of the course learning outcomes, ways to improve the course design in future offerings, and how the course could improve future career outcomes. A convenience sampling strategy was used for recruitment into the focus group session. A thematic content analysis was then conducted using the constant comparative method.

**Results:**

Major themes arising from student feedback were (1) appreciation of a customized patient case-based teaching approach, (2) more emphasis on using data analysis tools, (3) valuing interdisciplinary inclusion, and (4) request for more student discussion with advanced preparation materials.

**Conclusions:**

Feedback was generally positive and supports the continuation and expansion of the precision oncology course to include more hands-on instruction on the use of clinical bioinformatic tools.

**Supplementary Information:**

The online version contains supplementary material available at 10.1186/s12909-021-02500-6.

## Key messages

- A multi-disciplinary team of health center faculty is necessary to teach precision oncology.
- Biomedical and pharmacy students are willing to learn detailed clinical bioinformatic skills for the clinical application of precision oncology.

## Background

Oncologists use an enormous and growing amount of data to precisely diagnose and treat patients with cancer. For single cancer cell surface protein expressions such as human epidermal growth factor receptor 2 (HER2) or single cancer gene mutations such as *BCR-ABL* fusion, there are clear diagnostics and therapeutic guidelines for care [1, 2]. However, the majority of cancers are multigenetic and exhibit subclonal architecture [3, 4]. Moreover, results from oncology trials such as the SHIVA trial showed that therapies selected on the basis of single gene mutations failed to prolong survival in patients with metastatic cancers [5].

From the oncology clinician perspective, interpreting multi-biomarker tests results, such as those from next-generation sequencing (NGS), is challenging because of a lack of time and lack of expertise. Discussions from two recent National Academy of Medicine workshops identified the need for oncologists, pathologists, radiologists, bioinformatic engineers, geneticists, and molecular biologists to work more closely together in a multi-disciplinary team [6, 7]. Workshop participants recognized that tumor boards or precision oncology boards are a natural venue for this articulation of disciplines. Another recommendation from workshop participants was cross-training of professionals to speak each other’s language.

Molecular tumor boards and precision oncology decision support systems are increasingly being used for the optimal management of patients with cancer [8–10]. These precision boards and systems are made up of clinicians and scientists from multiple disciplines. A call for wider workforce knowledge and understanding of molecular medicine has been made [11]. However, there are neither standards nor experimental reports on how to train a workforce to participate or lead precision oncology boards or systems. Thus, we developed this graduate school course to prepare students to work on molecular tumor boards or other precision decision support systems. We purposely structured the training to include a multidisciplinary team of faculty, which mirrors the multidisciplinary structure of precision oncology boards.

## Methods

### Course design

We developed a one-credit hour (1 hour in-class instruction and a minimum of 2 hours homework per week), one semester (15 week) course for biomedical science graduate students (in the College of Medicine) and pharmacy graduate students (in the College of Pharmacy) enrolled in the University of Florida. The course was in-person and live. The learning objectives included: (1) interpret genomic test results in relation to the clinical management of cancer patients; (2) discuss patient genomic test results among clinical professionals; (3) explain the impact of contemporary genomic testing in the oncology clinic and describe opportunities for improvement; and (4) use online clinical bio-informatic tools for interpreting clinical genomic testing in patients with cancer. Course pre-requisites included having completed graduate level courses in cancer biology or human genetics, which exposed students to fundamentals of cancer-relevant data analytics. Course materials were made available via the University of Florida’s electronic learning management system and was accessible to students and faculty via password protected log-in. There was one in-person class per week with the option to videoconference for faculty or students. Five faculty were involved in the course. The faculty included a clinical oncologist, a clinical oncology pharmacists, a pharmacogeneticist, a genetic counselor, and a bio-informatician. The specific qualities for faculty included their first-hand experience with patients or clinical specimens, their ability to explain complex concepts clearly, and their patience to show students hands-on techniques for analyzing complex datasets. They engaged the students by asking them questions about the materials they presented or the science pertaining to the cases. The faculty also answered questions from the students. Each student presented one case. Patient visits were with select cases. For each class, two oncology cases were chosen by the oncology clinician, the primary instructor. Preferred case characteristics included cases of new cancer diagnoses, refractory cancer cases seeking consultation for treatment options, cases where pharmcogenotyping may influence drug dosing or choice, or cases when molecular diagnostic testing was available for monitoring disease response. Brief, de-identified summaries of the cases were posted on the course website approximately 5 days before the class. The electronic learning management system notified students and faculty immediately upon postings. Opportunities to immediately comment or ask questions were made available. However, the students and faculty were encouraged to collect their thoughts and bring them to share during the classes. When de-identified molecular data were presented from a cancer patient, it was customary for the faculty to introduce the type of data, its structure, methods for quality assurance and quality control, and standard procedures for data analyses.

After 3 weeks, students were assigned to lead case discussions following the format demonstrated by faculty during the first 3 weeks. During the class, the oncology clinician would orally present the case. Then the students would be invited to discuss the molecular and genomic features of the case based on background reading. Following this student presentation, the faculty would ask students about how to use that molecular and genomic data in determining the patient’s predisposition to cancer, cancer diagnosis, prognosis, treatment, and/or treatment response. When appropriate, faculty would demonstrate online tools and resources to answer these translational questions. After the class, the students were invited to visit with their assigned patient and the oncology clinician. During the patient visits, the students were asked to summarize for the patient the class discussions pertaining to their case. After the student-patient encounter, the oncology clinician would debrief with the student about the experience. Grading was based on in-class participation. Students were expected to participate in every class discussion by answering questions, asking questions, making comments, and relating material to other sources or experiences. If a student missed a class due to a personal emergency, then they were allowed to make up for their absence by submitting a one-page report on the topic of the missed class.

### Focus group

The study was approved by the University of Florida Institutional Review Board (#202000032). A focus group approach was chosen for data collection to capture the student learning community experience in context of the overall course framework of interprofessional training and to gather more exploratory, in-depth feedback on the potential impact to their future career and ways the course could be improved. On the last day of class (December 3, 2019), a 45-min focus group session was conducted by the instructional designer (HM), with no grading authority, using a semi-structured interview guide (Supplemental Digital Additional file 1) designed by the research team. The students were informed that their recorded responses would be kept confidential and analysis would be of an anonymized transcript, after final grades were submitted. Participation was optional and all five students in attendance (only one student was absent on this day) chose to participate.

The constant comparative method of coding data was used in a thematic analysis [12]. Two researchers (S.C., H.M.) collaborated over several meetings to discuss and manually code the entire data set and identify the emergent themes. For each main theme, we formed a conceptual definition and selected representative quotes. After the manuscript was drafted, the abstract and the results were sent to the students by electronic mail. Participants were invited (and sent two reminders over 3 weeks) to complete a survey where they could anonymously correct any unintentional misrepresentations of their viewpoints prior to publication [13]. Two students opted to respond directly to the communication instead of by questionnaire, both of whom agreed with our representation of their experience. Advantageously, one of these students was a biomedical graduate student and the other was from pharmacy so both disciplines were also represented in the member-checking.

## Results

Graduate students in biomedical sciences and pharmacy successfully completed the semester of classes with the multi-disciplinary team of faculty. Whereas, students attended each class in-person, occasionally faculty would connect via videoconference. In general, students were appreciative of the course and found it useful for their career. From the focus group, four major themes were identified and are presented in Table 1.

**Table 1:** Major Themes from Student Feedback (with example quotes)

**1. Appreciated Customized Patient Case-Based Teaching Approach**	
*Example Student Quotes:*	
1.1 “I thought it was neat, because for mine, we talking about test appropriateness. It was a good example of inappropriate test ordering and an over-utilization of that resource and then, also, not testing for the right genes and variants. So it was actually a really good example of what *not* to do, on the day I went with them to clinic. So sometimes it’s very helpful to see that.”	
1.2 “I thought of it as a … differential diagnosis and critical thinking exercise where [you] walk through- why order the test? What we would do if the results were X? And then, if the results are Y, what would we do? And then, sometimes, we talk about it and they were Z. So then it was just a whole [other] avenue that we didn’t entertain at first. And really, it’s almost like it was evolving throughout the semester, which was kind of fun. And it wasn’t scripted, it couldn’t have been scripted. It was a real patient.”	
1.3 “I’m in a translational research role or education career pathway … it’s really useful to see what the doctors think about and consider when doing everything. And I think that if I’m going to be successful at translating all this genetics research and actual clinical practice, then it’s really useful to have the perspective from the clinical side.”	
1.4 “I’m not usually in the clinic so, for me, it made the whole course just a lot more real … when you talk to the patient you have to make sure how you phrase things, and how they might not understand things the way you say it but then you have to rephrase it in simple words, and it was just interesting to me to see an actual patient in the clinic.”	
**2. More Genomic Data Analysis and Interpretation**	
*Example Student Quotes:*	
2.1 “I would have loved to, with one of the samples from one of the patients that we were looking at, if [the instructor] would’ve gone through the steps and how he put it into the program, how he got the data, what many manipulations he did. That is something that I would have found incredibly helpful … Even if it was extended by a longer period of time, I wouldn’t mind being here for two hours and seeing that example.”	
2.2 “I guess I’m not 100% clear on what the best databases are for looking at the variants.”	
2.3 “Some of the more basic translocations and stuff I knew, but some of the karyotyping we looked at here were pretty complex. That’s definitely a skill I learned walking away, being able to interpret those quickly.”	
**3. Precision Oncology Breadth Learned from Multidisciplinary Training**	
*Example Student Quotes:*	
3.1 “They used our input, which was nice. We discussed and we all came to a conclusion about what we should do with the patient. It was not just the experts discussing, being involved. Everyone who was part of the class and we all chipped in what we knew about the subject. That was nice, that we could be involved with that even though we are not doctors.”	
3.2 “I would have loved to see more of all [the other professionals] here at the same time … their discussions I found the most interesting and the part that I learned the most.”	
3.3 “I’ve been doing cancer research for a while, and now I’ve transitioned to regeneration. But during this class I’ve been able to identify where I would like to be participating further in my career, and the tools that are available, and the areas where we can expand and use towards understanding cancer better. And so it has really aided my research and my professional goals towards that area.”	
3.4 “I would say this was an actual true interdisciplinary course. Whereas other courses might throw that word in there, in their syllabus, this one genuinely was. So it was very much appreciated.”	
**4. Collaborative Learning Enhancement Ideas for Class Discussions**	
*Example Student Quotes:*	
4.1 “I think that he could have let us talk and flounder a little bit more before sort of revealing the answers. Sometimes I felt like I spent a lot of time reviewing the case and then he was basically like, ‘Okay it’s this.’”	
4.2 “Uploading the cases earlier would have been really helpful for me as well…[we] have to present part of the case, but we were given the assignment either the day of or the day before. And so there was very little time to adequately prepare for that.”	
4.3 “I’ve never specifically worked on Leukemias. I’ve done cancer research in the past, but I had enough background information to be able to pick it up…it would have been helpful to have just a collection of background reading posted on the website for people to get an intro if they needed it.”	

First, the students valued and enjoyed learning through active cases related to their research interests. In these case discussions they collaborated to interpret and evaluate data, as well as determined optimal clinical plans for improved patient care. The second major theme was a student request for more hands-on data analysis and interpretation. The students reported an understanding of current limitations of existing genetic testing. They quickly appreciated this and in the focus group asked for hands-on instruction in using informatic tools and online resources to conduct advanced genomic data analysis and interpretation, rather than a conceptual overview by faculty. Thirdly, the students appreciated and wanted more involvement from the multi-disciplinary faculty teaching them. They valued the insight provided from different professionals’ perspectives and envisioned future collaborations in their careers. Finally, students highly valued the discussion aspect of the course and asked for more opportunity to collaborate with each other. Specifically, they asked for less initial guidance by faculty and rearrangement of the room to facilitate collaborative problem-solving. Students also asked for more time to complete their background reading and prepare case presentations as well as supplemental materials for further exploration of areas they hadn’t yet encountered in their discipline. In the end, they identified the course as also being beneficial and recommended for their peers. One participant noted that with enrollment expansion, the course size should still be kept small (recommending 10–15 students) to maintain the value of small class discussions.

## Discussion

Students participating in this new graduate course gave positive feedback with critiques offered in the spirit of continuous quality improvement. Results from this study support the continuation of the course in generally the same format, emphasizing active cases with enhanced participation from the multidisciplinary instructor team. Feedback from the students (Theme 2) also support expansion of the course to include hands-on instruction on the use of clinical bioinformatic tools in the clinical application of precision oncology. One possibility is to offer the course as a two-credit course (doubling direct instruction to 2 hours of class time per week and 4 hours of homework time per week) with one of the lecture hours dedicated for hands-on training in how to analyze patient sequencing files. This instruction would be led by a clinical bioinformaticist and a molecular pathologist, and could follow the format of an existing course providing cross-disciplinary informatics training [14]. Another lecture hour could focus on the clinical interpretation and patient explanation of the data. This instruction would be led by the clinical faculty.

The focus group also suggested that future iterations of the course include more background material in the learning management system and provide more time for case review and preparation prior to the class session.

This course design is innovative in its use of multidisciplinary faculty to teach precision oncology to students across various health science professions. This is a culture shift for the college which historically has centers of excellence led by individual faculty members. However, as more professions hear from graduates about their real-world practice, there is a need to cross-train students so that they are prepared to work in multidisciplinary teams in their clinical practice.

From a faculty perspective, this graduate course was highly feasible and dovetailed with ongoing clinical care of cancer patients. Graduate programs accepting this kind of precision oncology course for degree requirements include those in biomedical sciences, pharmacy, medicine, or oncology. Tuition was charged for this course. Teaching effort was credited to participating faculty. The course did involve an electronic learning management system (Canvas, Instructure, Inc.) to organize course syllabus, materials, and communications. Cancer patients were universally receptive to voluntarily meet with the students and without remuneration.

As with any study using focus group methods, limitations can include difficulty in engaging participants, silencing minority perspectives, over-emphasizing perspectives from outspoken participants, and imposed bias from study investigators [11]. To mitigate against these limitations, we used a course facilitator who had no role in grading students, assurance of anonymous feedback with no possibility for retribution, and absence of all faculty members so that participants could speak freely. Furthermore, the course facilitator was skilled in facilitating group dialogue and keen to maintain an egalitarian space for discussion.

## Conclusions

In response to a need to increase the precision oncology workforce, we created a graduate school course that used multi-disciplinary, case-based, and hands-on learning of clinical bioinformatic and precision oncology skills. The course was well received by students, who called for even greater exposure to clinical bioinformatic coding and its clinical application in the precision oncology care of patients with cancer.

## Supplementary Information

**Additional file 1.**

## Data Availability

Not applicable.

## Abbreviations

BCR-ABL: Breakpoint cluster region – Abelson proto-oncogene 1
HER2: Human epidermal growth factor receptor 2
NGS: Next generation sequencing
