# Supplementary material for: Building a precision oncology workforce by multidisciplinary and case-based learning
Source: BMC Med Educ. 2021 Jan 26;21:75. doi: 10.1186/s12909-021-02500-6 (PMC7836489; doi:10.1186/s12909-021-02500-6)
Supplement: Supplementary file 1 — Additional file 1. [file 12909_2021_2500_MOESM1_ESM.docx]

# **Semi-Structured Interview Guide - Precision Oncology Course**

## Introduction to Students

Hi, I’m Dr. Heather Maness. I’m an Instructional Designer with UFIT’s Center for Instructional Technology and Training. As an Instructional Designer, I consult with faculty about their teaching and ways to improve student learning. The findings from this focus group session will be used to evaluate the effectiveness of this course as well as to make adjustments to improve future student experiences. Your openness and participation is highly encouraged but entirely voluntary. I believe there are no risks to you, nor are there benefits. With your permission I would like to record the session and share the anonymized transcript with Dr. Cogle and Dr. Chamala for analysis. I also ask that those participating also keep what was said during the session confidential. Are you ready to begin or would you like to ask a question before we get started?

## Questions

### General

1. How has this class helped prepare you for your career, if at all?
2. What elements of the course did you find most valuable to your learning?
3. What suggestions might you have for improving this course?
   - Is there another topic area that you feel should be represented or better represented in this course?
   - Is there another specialist’s perspective that you feel should be represented or better represented in this course?
   - Are there any skills or techniques that you feel should be included or could have been taught better?

### Course Goal Achievement

1. What could be done to improve current-day use of genomic testing and pharmacogenotyping in the oncology clinic?
2. Do you feel well-prepared and comfortable using online bioinformatics tools and resources for recommending and interpreting cancer genomic testing?
3. What was the impact of this course on your confidence and ability to interpret test results (such as chromosome karyotyping, next-generation sequencing, and complete blood counts) in relation to the clinical management of cancer patients?
4. Has this course made you more comfortable with approaching a clinical collaborator about creating and conducting a clinical research study?
5. If you met with a patient, how did that experience impact your learning precision oncology (or cancer genomics)?
   - Did this course make you more skilled or comfortable with talking to patients about their genomic test results?

### Student Composition/Peer Relationships

1. Did you find it beneficial to your learning to have students from both the College of Medicine and Pharmacy together in this course?
   - Did you ever interact together outside of class time?
   - Do you anticipate working on a project with one of your peers in the future?
2. Is there another degree program or student group that you think would benefit from offering this course as an elective to its students?

### Conclusion

1. Is there anything else that you would like to add?
